# A novel anti‐HIV‐1 bispecific bNAb‐lectin fusion protein engineered in a plant‐based transient expression system

**DOI:** 10.1111/pbi.13090

**Published:** 2019-03-12

**Authors:** Lauren E. Seber Kasinger, Matthew W. Dent, Garima Mahajan, Krystal Teasley Hamorsky, Nobuyuki Matoba

**Affiliations:** ^1^ James Graham Brown Cancer Center University of Louisville School of Medicine Louisville KY USA; ^2^ Department of Pharmacology and Toxicology University of Louisville School of Medicine Louisville KY USA; ^3^ Center for Predictive Medicine University of Louisville School of Medicine Louisville KY USA; ^4^ Department of Medicine University of Louisville School of Medicine Louisville KY USA

**Keywords:** broadly neutralizing antibody, high‐mannose‐type glycan, bispecific fusion protein, human immunodeficiency virus, plant virus vector, *Nicotiana benthamiana*

## Abstract

The discovery of broadly neutralizing antibodies (bNAbs) has been a major step towards better prophylactic and therapeutic agents against human immunodeficiency virus type 1 (HIV‐1). However, effective therapy will likely require a combination of anti‐HIV agents to avoid viral evasion. One possible solution to this problem is the creation of bispecific molecules that can concurrently target two vulnerable sites providing synergistic inhibitory effects. Here, we describe the production in plants and anti‐HIV activity of a novel bispecific fusion protein consisting of the antigen‐binding fragment (Fab) of the CD4 binding site‐specific bNAb VRC01 and the antiviral lectin Avaren, which targets the glycan shield of the HIV‐1 envelope (VRC01_Fab_‐Avaren). This combination was justified by a preliminary experiment demonstrating the synergistic HIV‐1 neutralization activity of VRC01 and Fc‐fused Avaren dimer (Avaren‐Fc). Using the GENEWARE^®^ tobacco mosaic virus vector, VRC01_Fab_‐Avaren was expressed in *Nicotiana benthamiana* and purified using a three‐step chromatography procedure. Surface plasmon resonance and ELISA demonstrated that both the Avaren and VRC01_Fab_ moieties retain their individual binding specificities. VRC01_Fab_‐Avaren demonstrated enhanced neutralizing activity against representative HIV‐1 strains from A, B and C clades, compared to equimolar combinations of VRC01_Fab_ and Avaren. Notably, VRC01_Fab_‐Avaren showed significantly stronger neutralizing effects than the bivalent parent molecules VRC01 IgG and Avaren‐Fc, with IC_50_ values ranging from 48 to 310 pm. These results support the continued development of bispecific anti‐HIV proteins based on Avaren and bNAbs, to which plant‐based transient overexpression systems will provide an efficient protein engineering and production platform.

## Introduction

The characterization of antibodies capable of neutralizing primary strains of human immunodeficiency virus type 1 (HIV‐1) in chronically infected individuals has greatly facilitated our understanding of the immune control of the virus and has opened the door for development of monoclonal antibodies as potential prophylactic and therapeutic agents. Some of these antibodies have impressive neutralization breadth and can elicit immune cell‐mediated cytotoxic responses against infected cells (Bruel *et al*., 2016). Several groundbreaking non‐human primate (NHP) and human studies have demonstrated that such broadly neutralizing antibodies (bNAbs) are effective at both preventing infection and decreasing viremia (Barouch *et al*., 2013; Bolton *et al*., 2016; Caskey *et al*., 2015; Gautam *et al*., 2016; Julg *et al*., 2017b; Lynch *et al*., 2015; Moldt *et al*., 2016). However, because a single bNAb is likely unable to overcome the great diversity of HIV‐1 and/or prevent the generation of escape mutations, therapy based on bNAbs will require the development of effective combination strategies. In fact, experiments in humanized mice and NHP infection models suggest that such a strategy may successfully control HIV‐1 infection in humans (Diskin *et al*., 2013; Julg *et al*., 2017a; Klein *et al*., 2012).

An optimal combination therapy would contain individual anti‐HIV‐1 agents that work together synergistically to inhibit the virus. However, finding bNAb combinations that produce more than additive effects has been difficult and optimal combinations are often strain dependent (Kong *et al*., 2015; Wagh *et al*., 2016). In contrast, bispecific antibodies capable of targeting two separate epitopes may exhibit strong synergy in HIV neutralization activity and thereby provide effective protection against infection *in vivo*. For instance, landmark studies by Bournazos *et al*. (2016); Huang *et al*. (2016) used CrossMab technology to generate bispecific neutralizing antibodies (biNAbs) that consisted of a combination of an bNAb and an anti‐CD4 or anti‐CCR5 antibody or a combination of two bNAbs respectively. Both groups were able to demonstrate that their most potent bispecific antibodies were able to neutralize over 90% of HIV‐1 strains tested and resulted in decreased viremia when administered to infected humanized mice. Despite their successes, it remains to be seen if these unique antibody designs are compatible with adequately producible, safe and pharmacologically viable profiles. Thus, the development of different classes of bispecific molecules may be justified.

A number of plant, algal and bacterial lectins have demonstrated anti‐HIV activity including cyanovirin‐N (CV‐N) (Boyd *et al*., 1997), griffithsin (Mori *et al*., 2005), concanavalin A (Matsui *et al*., 1990) and actinohivin (Chiba *et al*., 2001). These lectins interact with high‐mannose glycans (HMGs) found on the surface of some heavily glycosylated viral envelope proteins, like HIV gp120, allowing them to neutralize the virus by blocking its interaction with CD4 and coreceptors or aggregate virions (Boyd *et al*., 1997; Chiba *et al*., 2001; Mori *et al*., 2005). Lectins may have some benefits over bNAbs as antiviral agents. For example, viral resistance to lectins may cause a loss of glycosylation and thereby unmask previously hidden epitopes, making the virus potentially more vulnerable to the humoral immune system (Hu *et al*., 2007). Additionally, some lectins have broader specificity for viruses other than HIV (Helle *et al*., 2006; Meuleman *et al*., 2011; O'Keefe *et al*., 2003), providing opportunities for the development of prevention strategies against co‐infections.

The lectin actinohivin, isolated from actinomycete bacteria, is highly specific for HMGs and has excellent anti‐HIV activity but is difficult to produce recombinantly (Matoba *et al*., 2010). Recently, we created a variant of actinohivin called Avaren, which showed greatly improved producibility in a plant expression system (Hamorsky *et al*., 2019; submitted; Matoba *et al*., 2014). Notably, Avaren fused to the fragment crystallizable region (Fc) of human IgG1 (Avaren‐Fc; dimerized through the Fc domain) was expressed at a very high level upon transient overexpression in *Nicotiana benthamiana* and showed cross‐clade neutralizing activity against 20 primary HIV‐1 viruses and HIV‐2 strains, with a median IC_50_ of 0.3 nm (Hamorsky *et al*., 2019; submitted; Matoba *et al*., 2010). These results provide a basis for the development of novel bispecific HIV inhibitors based on Avaren and bNAbs. In this work, we describe the production and anti‐HIV activity of one such bispecific entry inhibitor composed of a fusion of Avaren to the Fab portion of the CD4 binding site‐specific bNAb VRC01 (Li *et al*., 2011) to create VRC01_Fab_‐Avaren. VRC01 is one of the most well‐studied bNAbs, and has previously been shown to be safe and effective in humans (Ledgerwood *et al*., 2015; Lynch *et al*., 2015). Furthermore, it has previously been effectively expressed to a high degree in *N. benthamiana* by us and other groups (Hamorsky *et al*., 2019; submitted; Rosenberg *et al*., 2013; Teh *et al*., 2014). To produce this molecule, we employed the GENEWARE^®^ transient expression vector derived from the 30B tobacco mosaic virus (TMV) vector (Shivprasad *et al*., 1999), which allows for the robust production of foreign proteins in *N. benthamiana* leaf tissue. Plant expression systems offer enhanced production speed, scalability and safety compared to conventional cell‐culture based methods making them an attractive option for pharmaceutical protein production. The work presented herein demonstrates further that antiviral lectins can partner with bNAbs for the generation of potent bispecific anti‐HIV‐1 agents and that VRC01_Fab_‐Avaren provides a prototype for the development of more effective bi‐ and trispecific inhibitors.

## Results

### Synergy of Avaren‐Fc and VRC01

**Figure 1:**
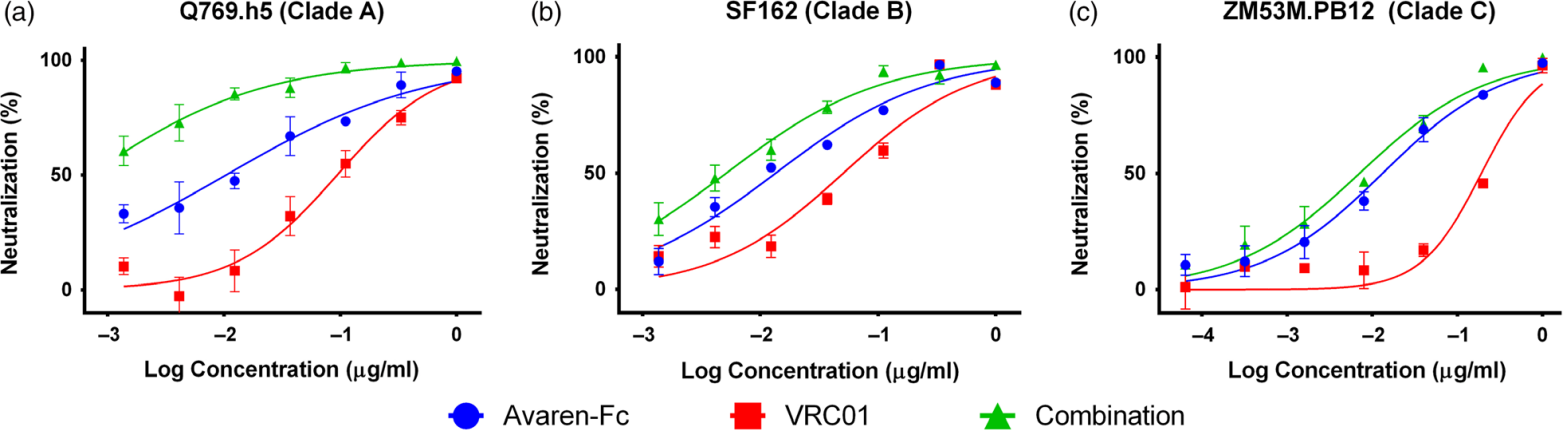
Anti‐HIV synergy between Avaren‐Fc and VRC01. Synergy was shown in three different HIV‐1 virus strains using an Env‐pseudotyped HIV‐1 neutralization assay. (a) Q769.h5, clade A. (b) SF162, clade B. (c) ZM53M.PB12, clade C. CalcuSyn software (Biosoft) was used to determine the degree of synergism between Avaren‐Fc and VRC01 based on the multiple‐drug effect equation. Mean combination index (CI) values at IC_50_ levels were 0.25 ± 0.07 (Q769.h5), 0.62 ± 0.13 (SF162) and 0.30 ± 0.08 (ZM53M.PB12), summarized in Table S1. A CI of <0.9 indicates synergy, 0.9–1.1 indicates addition, and >1.1 indicates antagonism.

Among different bNAbs, VRC01 was selected as a candidate for fusion with Avaren because it is one of the most well‐studied bNAbs for HIV‐1 and has demonstrated safety and efficacy in humans. To test the compatibility of VRC01‐Avaren combination, we investigated what type of effect (synergistic, antagonistic or additive) these proteins had in combination when attempting to neutralize HIV. To test this interaction, we employed an Env‐pseudotyped HIV‐1 neutralization assay using HOS‐CD4‐CCR5+ cells. Avaren‐Fc and VRC01 IgG were tested alone and in combination; Avaren‐Fc was used so that the two anti‐HIV proteins are evaluated for combinatorial effects under equivalent (i.e. bivalent dimer) conditions. Individually, both molecules exhibit strain‐specific low nanomolar neutralization activity. To test combinatorial effects in a wider breadth of viruses, three strains (Q769.h5, SF162 and ZM53M.PB12) spanning three HIV‐1 clades (A, B and C) were tested. In the assays, Avaren‐Fc and VRC01 were first mixed together and then diluted to maintain a constant ratio between the two at each concentration. The combinations of Avaren‐Fc and VRC01 displayed synergism in all HIV‐1 strains tested as shown by combination index (CI) values less than 1 (Figure 1, Table S1). Since the combination of Avaren‐Fc and VRC01 was superior to either drug alone in all tested strains spanning three HIV‐1 clades, we concluded that production and characterization of a fusion consisting of the two proteins would be warranted.

### Expression and purification of the fusion, VRC01_Fab_‐Avaren

**Figure 2:**
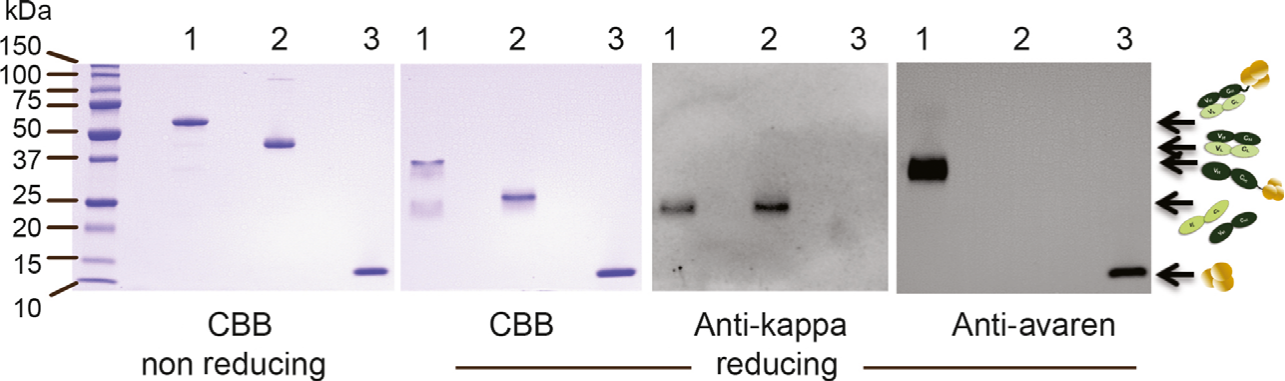
Expression and purification of VRC01_Fab_‐Avaren fusion along with the monomer species, VRC01_Fab_ and Avaren, in *Nicotiana benthamiana*. CBB‐stained SDS‐PAGE with protein separated under non‐reducing and reducing conditions (far left and middle left panels respectively) in conjunction with anti‐kappa light chain and anti‐Avaren immunoblots (middle right and far right panels respectively). This figure shows not only the high purity of each protein purified but also the composition of VRC01_Fab_‐Avaren as the Fab heavy chain covalently linked to Avaren. In contrast, the VRC01_Fab_ consists of the heavy and light chains at 25 kD. Each lane contains the same sample in each panel. Lane 1: VRC01_Fab_‐Avaren. Lane 2: VRC01_Fab_. Lane 3: Avaren.

To test the feasibility of fusions of VRC01 and Avaren, we constructed a simple prototype protein consisting of one Fab molecule fused to one Avaren molecule, in which Avaren was attached to the C‐terminal of the heavy chain of VRC01_Fab_ by a glycine–serine linker. The fusion protein was produced from a single transgene in *N. benthamiana* using the TMV vector system GENEWARE^®^. This transgene contained the subtilisin‐like processing protease kex2p positioned between the heavy and light chains of the Fab whereby during expression the heavy and light chain would be translated as a single polypeptide followed by separation in the trans‐Golgi via cleavage by the kex2p protease. Once cleaved, the heavy and light chains would assemble into a functional Fab (Hamorsky *et al*., 2019; submitted). The overexpressed VRC01_Fab_‐Avaren fusion was then extracted from the plants and purified using a three‐step chromatography procedure. To display the purity and identity of our fusion protein, CBB‐stained SDS‐PAGE and Western blots probing for Avaren and the kappa light chain of the Fab were performed on the VRC01_Fab_‐Avaren fusion (Figure 2, lane 1), Fab (Figure 2, lane 2) and Avaren (Figure 2, lane 3). VRC01_Fab_‐Avaren has a molecular weight of 61.8 kDa, with the VRC01_Fab_ portion comprising 49 kDa and the Avaren domain the remaining 13 kDa. These theoretical molecular sizes were confirmed in SDS‐PAGE (Figure 2, far left panel) under non‐reducing conditions. When the samples were analysed in reducing conditions (Figure 2, middle left panel), two bands were present in the VRC01_Fab_‐Avaren sample (Figure 2, lane 1) which is the evidence of the heavy chain and light chain separating from reduction of the joining disulfide bond. The heavy chain with the covalently attached Avaren ran higher (≈37 kDa) than the light chain (25 kDa) under these reducing conditions. In contrast, VRC01_Fab_ (Figure 2, lane 2) ran as only one band because the heavy and light chains have similar molecular weights (25 kDa each).

To further confirm that Avaren was linked to the Fab heavy chain in the fusion, a Western blot probed with anti‐Avaren guinea pig serum (Figure 2, far right panel) was performed. This yielded a band at an reasonable size (≈37 kDa) for heavy chain‐Avaren. Another blot was performed and probed with anti‐ human kappa antibody (Figure 2, middle right panel) to detect the light chain of VRC01_Fab_ in the fusion (Figure 2, lane 1) and alone (Figure 2, lane 2). One band did appear on the blot for the fusion and VRC01_Fab_ alone, each at 25 kDa, an appropriate size for light chain Fab. Taken together, the results demonstrate the identity of VRC01_Fab_‐Avaren on the basis of molecular weight size and reactivity with specific antibodies. The SDS‐PAGE result also shows the purity of VRC01_Fab_‐Avaren, VRC01_Fab_ and Avaren to be >95% as determined by densitometry analysis (Figure 2, far left panel), which were used in other experiments presented here.

### Confirmation of bispecificity of VRC01_Fab_‐Avaren

**Figure 3:**
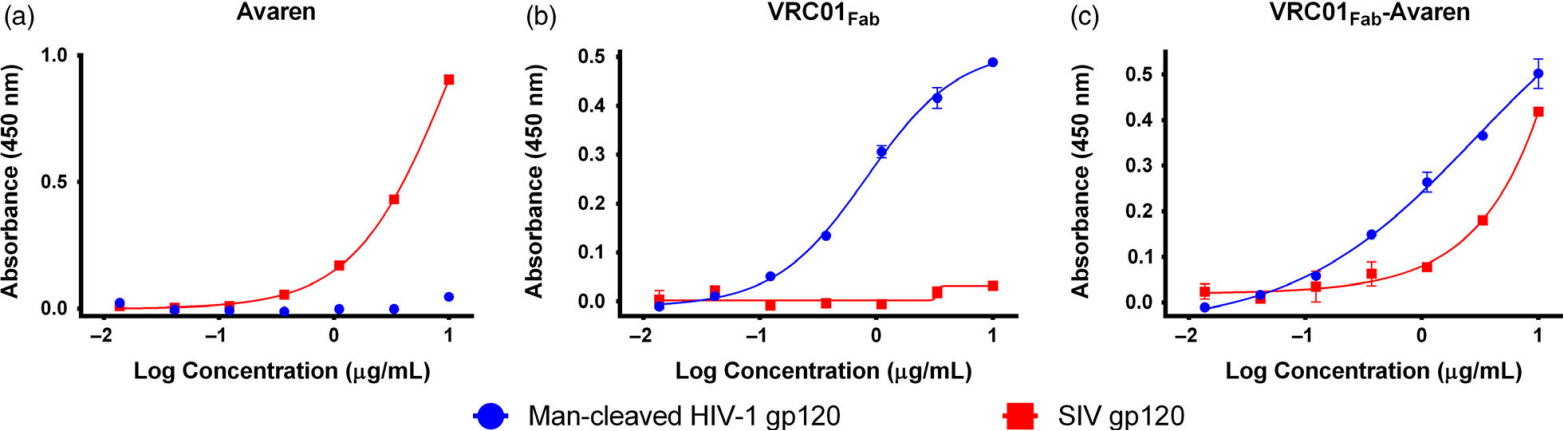
Confirmation of the bispecificity of VRC01_Fab_‐Avaren with gp120 ELISA. ELISA was used to qualitatively demonstrate the bispecificity of VRC01_Fab_‐Avaren. The 96 well plates were coated with gp120 from mannosidase‐cleaved SF162 strain HIV‐1 or SIVmac239 to confirm the bispecificity of VRC01_Fab_‐Avaren. (a) Since SIV gp120 is also coated with HMGs, Avaren is capable of binding to SIV gp120 but VRC01 is not. (b) VRC01_Fab_ is capable of binding to mannosidase‐cleaved gp120 from strain SF162 but cannot bind to SIV gp120. (c) VRC01_Fab_‐Avaren is capable of binding to both mannosidase‐cleaved SF162 gp120 and SIV gp120, demonstrating the bispecificity of the molecule.

Successful expression and purification of VRC01_Fab_‐Avaren prompted us to demonstrate the functionality of each portion of the fusion molecule. In particular, it is important to ensure that the artificial linkage of the two proteins does not compromise the specific binding activity of either constituent. Due to the overlapping nature of both Avaren and VRC01_Fab_'s target (both bind HIV‐1 gp120), two distinctive gp120‐capture ELISA methods were developed, by which the binding specificity and affinity of the two respective fusion domains were characterized. The first ELISA utilized a gp120 from simian immunodeficiency virus (SIV), taking advantage of the fact that VRC01_Fab_ is specific for the CD4‐binding site of HIV‐1 gp120 (Li *et al*., 2011). In contrast, Avaren binds to HMGs, which are present on both HIV‐1 and SIV gp120s. As shown by dose–response curves in graphs of Figure 3a–c, dose‐dependent binding was observed with Avaren (Figure 3a) and VRC01_Fab_‐Avaren (Figure 3c) between 0.1 and 10 μg/mL in the SIV gp120 ELISA, while VRC01_Fab_ did not show any significant binding in the concentration range analysed (Figure 3b). The second ELISA utilized a mannosidase‐treated HIV gp120 (SF162, clade B). Given that Avaren likely binds to the terminal mannose residues of HMGs on the surface of gp120 (Hamorsky *et al*., submitted; Matoba *et al*., 2014), mannosidase treatment would eliminate Avaren's ability to bind gp120. On the contrary, VRC01_Fab_ binds to the CD4‐binding site and thus should bind gp120 regardless of mannosidase treatment. As predicted, Avaren was unable to bind mannosidase‐treated gp120 (Figure 3a) while both VRC01_Fab_ (Figure 3b) and the fusion (Figure 3c) showed clear dose‐dependent binding. Coupled together, the ELISA results indicate that both Avaren and VRC01_Fab_ moieties of the fusion protein retain binding activity to their respective targets.

**Figure 4:**
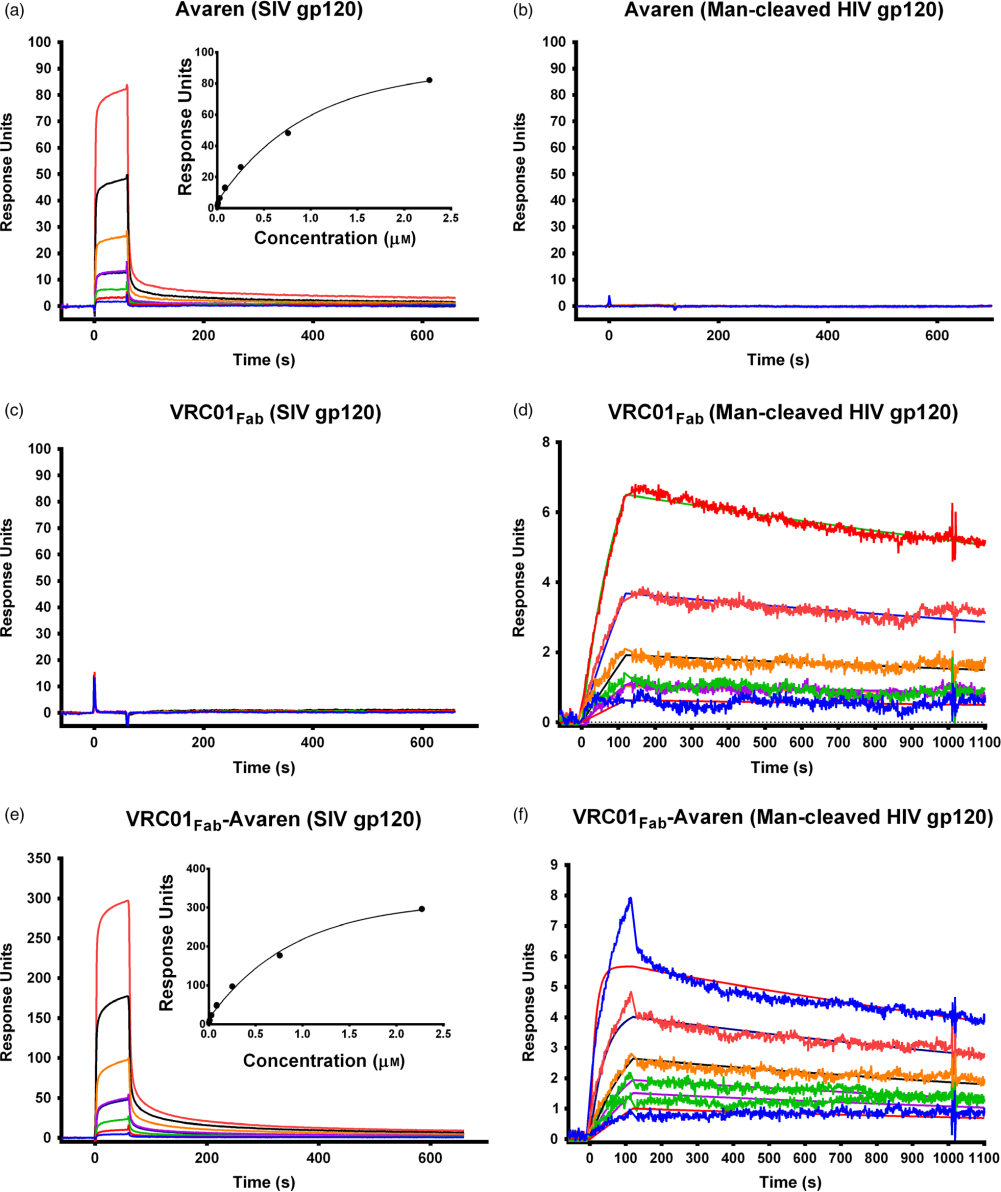
Confirmation of the bispecificity of VRC01_Fab_‐Avaren with SPR. SPR was used to quantitatively determine the bispecificity of VRC01_Fab_‐Avaren. (a) Avaren, (c) VRC01_Fab_ and (e) VRC01_Fab_‐Avaren were flowed over a sensor chip immobilized with biotinylated SIVmac239 gp120 via streptavidin. The curves shown in the sensorgrams represent the response of several threefold dilutions of each analyte: Avaren beginning at 2.5 μm, VRC01_Fab_ beginning at 740 nm/mL and VRC01_Fab_‐Avaren beginning at 3.7 μm shown from top to bottom. For each protein, the experiment was performed in duplicate. Similarly, (b) Avaren, (d) VRC01_Fab_‐Avaren and (f) VRC01_Fab_‐Avaren were flowed over a sensor chip immobilized with biotinylated mannosidase‐cleaved SF162 gp120 via streptavidin. The curves shown in the sensorgrams represent the response of several threefold dilutions of each analyte: Avaren and VRC01_Fab_ beginning at 333 nm and VRC01_Fab_‐Avaren beginning at 400 nm shown from top to bottom. For each protein, the experiment was performed in duplicate. The measured affinities for each experiment are summarized in Table 1.

To substantiate the bispecificity further and determine the binding affinity of each moiety of VRC01_Fab_‐Avaren, surface plasmon resonance (SPR) experiments were performed. These experiments mirrored the ELISA experiments by using the same gp120 targets, from SIV‐ and mannosidase‐treated HIV‐1 SF162. To faciliate the direct comparison of binding affinities among Avaren, VRC01_Fab_ and VRC01_Fab_‐Avaren, each gp120 target was immobilized to a streptavidin‐coated sensor chip through a biotin tag, to which the analytes (Avaren, VRC01_Fab_, or VRC01_Fab_‐Avaren) were flowed over the sensor chip. As shown in Figure 4a,e, Avaren and the fusion protein bound to SIV gp120 with an average equilibrium dissociation constant (*K*_D_) of 1.09 and 1.04 μm, respectively, while VRC01_Fab_ showed no detectable binding (Figure 4c). No statistical difference was found between the *K*_D_ values of Avaren and VRC01_Fab_‐Avaren in an unpaired Student's *t*‐test. These results strongly suggest that the Avaren moiety of the fusion molecule retains its full binding capacity to HMGs on gp120. Meanwhile, VRC01_Fab_ and VRC01_Fab_‐Avaren are bound to mannosidase‐treated gp120 with a *K*_D_ of 8.55 and 2.23 nm, respectively, whereas Avaren failed to show measurable binding (Figure 4b,d, f). Although the affinities of VRC01_Fab_ and VRC01_Fab_‐Avaren were statistically different (*P* = 0.037; unpaired *t*‐test), the fact that Avaren did not display any binding to the mannose‐cleaved gp120 suggests that the Fab domain of the fusion protein maintains its functionality. The *K*_D_ values are summarized in Table 1. In conclusion, SPR showed that Avaren and VRC01_Fab_ moieties in the fusion molecule largely retained binding affinity to their respective target, further validating the feasibility of VRC01_Fab_‐Avaren as a prototype bispecific anti‐HIV‐1 agent.

**Table 1:** Comparison of the affinities of VRC01_Fab_‐Avaren and parental monovalent species to SIV gp120 or mannose‐cleaved HIV gp120

Molecule	Dissociation constants (*K*_D_, μm ± SEM)	
	SIV gp120	Man‐cleaved HIV gp120
Avaren	1.09 ± 0.01	N/D
VRC01_Fab_	N/D	8.55 ± 1.20
VRC01_Fab_‐Avaren	1.04 ± 0.04	2.23 ± 0.23

### HIV‐1 neutralization activity of VRC01_Fab_‐Avaren

**Figure 5:**
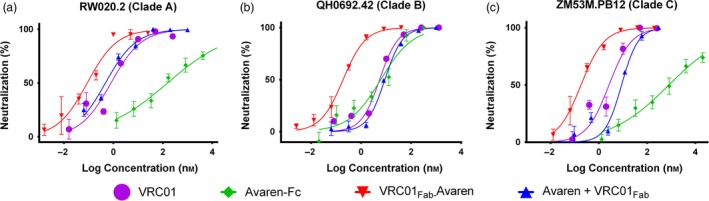
HIV neutralization of VRC01_Fab_‐Avaren and its parental monovalent and bivalent species. An Env‐pseudotyped HIV‐1 neutralization assay was used to assess the activity of VRC01_Fab_‐Avaren as compared to its parental monovalent and bivalent species (Avaren, VRC01_Fab_, Avaren‐Fc, and VRC01) as well as a mixture of Avaren and VRC01_Fab_ against three viruses spanning three clades: (a) RW020.2 from clade A, (b) QH0692.42 from clade B and (c) ZM53M.PB12 from clade C. The IC_50_ values for each molecule are summarized in Table 2. Overall, VRC01_Fab_‐Avaren showed greater neutralizing activity than any of the parent species, including an equimolar mixture of both Avaren and VRC01_Fab_.

To determine the anti‐HIV‐1 activity of VRC01_Fab_‐Avaren, an envelope (Env)‐pseudotyped HIV‐1 neutralization assay was performed using three R5 strains of HIV‐1 spanning A, B and C clades (RW020.2 clade A, QH0692.42 clade B and ZM53M.PB12 clade C). As shown in Figure 5a–c, VRC01_Fab_‐Avaren showed potent and dose‐dependent neutralizing effects against all three viruses with near‐complete inhibition obtained <10 nm. Notably, the fusion molecule showed superior potency when compared to a 1 : 1 equimolar mixture of the parental monomers, VRC01_Fab_ and Avaren, in all three strains tested, despite that the only difference between these two treatments is the covalent linkage in the fusion. Furthermore, VRC01_Fab_‐Avaren showed superior potency when tested against parental dimers, Avaren‐Fc and VRC01 in all three viruses tested. Table 2 summarizes the 50 per cent inhibitory concentration (IC_50_) values of VRC01_Fab_‐Avaren, 1 : 1 equimolar mixure of VRC01_Fab_ and Avaren, Avaren‐Fc and VRC01 in the HIV‐1 neutralization assays. The IC_50_ values of VRC01_Fab_‐Avaren were lower than those of 1 : 1 equimolar mixure of parental monomer molecules or each of their bivalent dimer forms in all three viruses tested. The fusion protien exhibited a 23‐fold lower, a 61‐fold lower and a 30‐fold lower IC_50_ than the equimolar mixture of VRC01_Fab_ and Avaren in RW020.2 A clade virus, QH0692.42 B clade virus and ZM53M.PB12 C clade virus respectively.

**Table 2:** HIV neutralization activity VRC01_Fab_‐Avaren compared to parental monovalent species and bivalent species

Molecule	Mean IC_50_ ± SEM (nm)		
	RW020.2 (Clade A)	QH0692.42 (Clade B)	ZM53M.PB12 (Clade C)
Avaren	>1000	>1000	>3000
Avaren‐Fc	194.1 ± 39.72	6.01 ± 2.74	777.7 ± 106.4
VRC01_Fab_	1.63 ± 0.59	12.60 ± 4.58	7.54 ± 1.95
VRC01	0.59 ± 0.59	5.16 ± 0.18	2.93 ± 0.47
Avaren + VRC01_Fab_	1.11 ± 0.48	10.75 ± 3.66	9.18 ± 2.67
VRC01_Fab_‐Avaren	0.05 ± 0.02	0.17 ± 0.01	0.31 ± 0.11

## Discussion

It is well‐known that the most effective methods of treating HIV infection involve the use of combination treatments because the wide diversity and high mutation rate of the virus allows it to escape monotherapy rather quickly (de Mendoza, 2016). The use of a bispecific molecule pursuing two different targets on the virus may provide increased ability to overcome HIV's diversity. In this paper, we engineered a novel bispecific molecule based on the CD4 binding site‐specific bNAb VRC01 and the antiviral lectin Avaren, which showed superior potency compared to its parental proteins. The fusion protein, VRC01_Fab_‐Avaren, was expressed in *N. benthamiana* at approximately 40 mg/kg of leaf biomass, which was lower than those achieved for the parental molecules (~150 mg/kg for VRC01 (Matoba *et al*., 2014; Hamorsky *et al*., submitted), and over 100 mg/kg for Avaren). The yield may be improved by further optimization of expression and/or purification conditions, thereby making it more viable for scale up and manufacturing. The synergism displayed in the mixture of the bivalent parental molecules VRC01 IgG and Avaren‐Fc (Figure 1), both of which we have previously produced in *N. benthamiana* (Matoba *et al*., 2014; Hamorsky *et al*., submitted), provided a rationale for, and strengthened our confidence in, utilizing these molecules in a fusion. To obtain a proof of concept, we opted for a simpler approach in this foundational study, creating a bispecific molecule monovalent for each antigen, VRC01_Fab_‐Avaren.

Using three chromatography steps, we purified VRC01_Fab_‐Avaren and confirmed each constituent's identity by Western blot analysis (Figure 2) and functionality through ELISA (Figure 3) and SPR (Figure 4). We successfully uncovered and characterized the binding activity of individual components of the bispecific molecule with the ELISA and SPR analyses. Under the conditions in which the ability of one of the fusion's constituents is removed, the ELISA binding curves and SPR sensorgram of the fusion generally matched those of the active constituent alone. Avaren, for instance, is only capable of binding to gp120 when the HMGs on the surface are present, as in the case of SIV gp120 in our assay system. On the other hand, VRC01 is specific for gp120 derived from HIV. By using SIV gp120 and mannose‐cleaved HIV gp120, the distinction between the binding activity of VRC01_Fab_ and Avaren can be made, confirming the bispecificity of the fusion molecule which has activity against both forms (Figure 3). Further confirmation was provided by SPR analysis using the same experimental approach, with Avaren binding only to SIV gp120 with a similar affinity as the fusion and VRC01_Fab_ binding only to the mannose‐cleaved HIV gp120. Interestingly, one exception was observed in the SPR analysis using mannosidase‐treated HIV‐1 gp120, in which the *K*_D_ value for VRC01_Fab_‐Avaren was nearly fourfold better than VRC01_Fab_ alone (2.2 vs. 8.5 μm). This may be attributed to the incomplete enzymatic digestion of the glycans on SF162 gp120. We speculate that a few remaining glycans, which were apparently insufficient for the Avaren monomer to bind to with any measurable affinity (Figure 4), could have been sufficient for the Avaren moiety to bind when the other moiety (i.e. VRC01_Fab_) in the fusion docked into the CD4 binding site. Nevertheless, these results along with those from ELISA demonstrate that each constituent in the fusion was active and not negatively affected by the covalent linkage in the context of VRC01_Fab_‐Avaren.

In all HIV viruses tested here, spanning three major clades circulating in the world (Buonaguro *et al*., 2007), VRC01_Fab_‐Avaren exhibited superior potency compared to the monomer parental molecules, Avaren and VRC01_Fab_, and the bivalent parental molecules, Avaren‐Fc and VRC01 IgG, displaying IC_50_ values in the mid to low picomolar range (Figure 5, Table 2). It is worth noting that the fusion was between two‐ and 30‐fold more potent than VRC01 alone and between 34‐ and >4000‐fold more potent than Avaren‐Fc alone, even though all three molecules have bivalent binding capacity. This illustrates the fundamental advantage of bispecific targeting over monospecific inhibition against HIV‐1 infection. Of another interest is that VRC01_Fab_‐Avaren showed significantly better IC_50_ values than an equimolar mixture of the two fusion components. This side‐by‐side comparison allowed us to see the vital effect that covalently linking these two molecules together had on its anti‐HIV potential.

The mechanism by which these fusion proteins exhibit such strong neutralization activity is unknown. In Figure S1, the SPR sensorgram (fit with a 1 : 1 binding model with *R*_max_ set to local) shows that Avaren (Figure S1A) associates and dissociates faster than VRC01_Fab_ (Figure S1B), though the two molecules have similar affinities to gp120 (inset table). This seems to indicate that Avaren and VRC01_Fab_ have distinct modes of interaction with HIV‐1, which may provide clues as to why the fusion was more potent *in vitro* than an equimolar mixture of VRC01_Fab_ and Avaren. For instance, the initial binding of the Avaren portion of the fusion protein to Env glycans may concentrate the VRC01_Fab_ moiety on the surface of the virus at the point of contact, thus allowing it to neutralize more effectively. A similar phenomenon has been hypothesized to occur with the bispecific mAb 10E8/iMab, which may concentrate the bNAb 10E8 domain on Env spikes via iMab's binding to the CD4 receptor, greatly improving its activity (Huang *et al*., 2016). Upon visual inspection, the sensorgram for VRC01_Fab_‐Avaren (Figure S1C) does seem to show bivalent binding, with both slow and fast association and dissociation components. Fitting the curve to a bivalent model, however, did not give reliable rate and dissociation constants, with highly non‐random residual plots possibly due to steric hindrance caused by the high density of gp120 on the surface of the chip. Fitting to the 1 : 1 model gave constants that seemed to more accurately represent the curve, though the fit may be improved by using a lower density chip to limit bivalent interactions. According to this model, the fusion of Avaren to VRC01_Fab_ improves the overall affinity three to fivefold (Figure S1, inset table), which may partly explain the significant improvement in the neutralization capacity. However, it is important to note that fast analyte–ligand interactions observed with Avaren and the fusion (Figure S1A,C) may be interpreted as bulk refractive index (RI) changes, making the rate constant calculations less accurate and complicating this hypothesis. Another caveat of the SPR experiment in the present study is the nature of the ligand (i.e. gp120) used in the analysis; whereas we immobilized recombinant monomeric gp120 proteins on the sensor chip, native gp120 trimerizes into spikes on HIV‐1 envelope. Thus, further studies characterizing inter‐ and intra‐spike binding as well as binding stoichiometry between VRC01_Fab_‐Avaren and native Env are necessary to scrutinize the fusion protein's HIV‐1 inhibitory mechanism.

A number of bispecific anti‐HIV agents have been produced in the last decade. The most prominent examples of these are the biNAbs produced by Bournazos *et al*. (2016); Huang *et al*. (2016), which consist of a combination of a bNAb and an anti‐CD4 antibody or a combination of synergistic bNAbs respectively. Both of these biNAbs were able to neutralize the vast majority of strains tested with IC_50_ values in the pm range and were rationally designed after testing several combinations of antibodies to find synergistic pairs. A similar approach to our group's was previously used in 2009 when Sexton *et al*. (2009) demonstrated the expression *in planta* and the anti‐HIV activity of a fusion of the bNAb b12 and the HMG‐binding lectin CV‐N. Unlike VRC01_Fab_‐Avaren, this fusion consisted of the full‐length b12 mAb fused at the C‐terminus to CV‐N and was produced in transgenic plants. While it was capable of neutralizing the virus, little else can be said about the molecule's properties since it was not purified and characterized. Furthermore, it has been demonstrated that the use of CV‐N, and lectins in general, may be limited due to its potential cytotoxicity and mitogenicity (Balzarini *et al*., 2006). Avaren, on the other hand, has not been shown to be mitogenic or cytotoxic (Matoba *et al*., 2010). Lastly, while studies have established the efficacy of b12 in animal models, VRC01 has been shown to have greater neutralization breadth and has been demonstrated to be safe and efficacious in humans (Ledgerwood *et al*., 2015; Lynch *et al*., 2015; Parren *et al*., 2001). These facts combined with the high degree of producibility in a plant system demonstrate that VRC01_Fab_‐Avaren has strong potential as a possible anti‐HIV agent.

One possible way to improve the efficacy of a bispecific molecule like VRC01_Fab_‐Avaren may be to optimize the length of the linker between VRC01_Fab_ and Avaren moieties to increase intra‐Env spike binding activity. It has been demonstrated previously that HIV may evade neutralizing antibodies by forcing them to bind monovalently to gp120 (Klein and Bjorkman, 2010; Mouquet *et al*., 2010). Additionally, the relatively low density of gp160 trimers on the surface of the virus may also hinder inter‐spike binding (Schiller and Chackerian, 2014; Zhu *et al*., 2006). According to Galimidi *et al*. (2015), who dissected the effect of linker length on the neutralizing activity of two bNAb Fab domains, the optimum length to facilitate intra‐spike binding and improve neutralization activity is approximately 211 Å. Conversely, the GGGGSGGGGS linker employed here to link VRC01_Fab_ to Avaren is only approximately 35 Å. Thus, it may be possible to improve the neutralizing activity of VRC01_Fab_‐Avaren by increasing the length of the linker. This is not without its own pitfalls, as an optimal linker length and composition would need to be determined and without optimization, the stability and producibility of the molecule may be compromised (Chen *et al*., 2013). However, the ease and speed at which recombinant proteins can be produced in plant‐based transient overexpression systems is such that many different linker lengths and compositions could be tested in a rather short period of time.

In summary, we have shown that the novel bispecific fusion molecule, VRC01_Fab_‐Avaren, displays increased potency *in vitro* against HIV‐1 by combining the distinctive inhibitory mechanisms of a bNAb specific for the CD4‐binding site on gp120 and a lectin specific for HMGs on the surface of gp120. With the engineering of new, more highly potent bNAbs (Diskin *et al*., 2011), our unique bispecific fusion approach demonstrated here could be used as a platform to test fusions containing different bNAbs_._ The use of a plant‐based transient expression system also will contribute to the speed at which these various fusions can be produced for characterization. As an effective HIV vaccine has yet to become a reality, VRC01_Fab_‐Avaren may add to the repertoire of anti‐HIV molecules to be used for prevention or treatment of HIV.

## Experimental procedures

### Vector construction of VRC01_Fab_‐Avaren and expression in *N. benthamiana* using GENEWARE^®^

The gene sequence for VRC01_Fab_‐Avaren was designed *in silico* by linking VRC01_Fab_ to Avaren with a glycine linker (GGGGSGGGGS) and synthesized (ATUM, Newark, CA, USA). For use in plants, the restriction enzymes PacI and AvrII were used to transfer the synthesized gene into the GENEWARE^®^ vector (Kentucky Bioprocessing, LLC, Owensboro, KY, USA). For infection of *N. benthamiana*, infectious RNA was produced using the mMessage mMachine^®^ T7 Transcription Kit from Life Technologies (Waltham, MA, USA). The transcription reaction was performed according to the manufacturer's instructions, using 1 μg of plasmid DNA template and including an extra microlitre of GTP to improve large transcript efficiency. To confirm a successful reaction, 2 μL of the product was visualized by agarose gel electrophoresis. The method for inoculating *N. benthamiana* plants with infectious transcript was followed as outlined in Moore *et al*. (2016). Plants were then incubated in a 23 °C humidified growth chamber for 11 days after inoculation and the tissue was harvested.

### Extraction and purification of VRC01_Fab_‐Avaren

Fresh, harvested tissue was homogenized with ice‐cold extraction buffer (20 mm Sodium Citrate, 40 mm Ascorbic Acid, pH 4) in a 2 : 1 buffer to biomass ratio using a Waring blender. The homogenized extract was filtered through four layers of cheesecloth and one layer of miracloth prior to centrifugation at 15 000 *g* for 15 min at 4 °C. The supernatant pH was adjusted to 6 and centrifuged again before being filtered through a 0.22‐μm polyethersulfone (PES) filter. Purification was performed in three steps, beginning with anion exchange chromatography with SP Fast Flow Resin (GE Healthcare, Chicago, IL, USA), by multimodal chromatography with a 5 mL HiTrap Capto MMC column (GE Healthcare), and finally with a 5 mL CHT Type I 40 μm resin (Bio‐Rad Laboratories, Hercules, CA, USA).

Prior to loading the sample, the 40 mL SP column (2.6 cm × 7.5 cm) was equilibrated with 10 column volumes (CV) of SP Buffer A (20 mm Sodium Citrate buffer, pH 6) at a flow rate of 20 mL/min. The sample was loaded onto the column at 15 mL/min. Then, the column was washed with 10 CV of Buffer A to remove all unbound proteins. To elute VRC01_Fab_‐Avaren, a linear gradient from 0 to 1 m NaCl utilizing both SP Buffer A and SP Buffer B (20 mm Sodium Citrate buffer + 1 m NaCl, pH 6) over 20 CV was performed at 20 mL/min and collected in 40 mL fractions. The VRC01_Fab_‐Avaren ‐containing fractions (eluted between 10% Buffer B and 25% Buffer B) were pooled for loading onto the MMC column.

The 5 mL MMC column was equilibrated with 10 CV of MMC Buffer A (20 mm Sodium Citrate buffer + 500 mm NaCl, pH 6) at a flow rate of 5 mL/min, with pooled sample from the previous chromatography step loaded onto the column at 2.5 mL/min. VRC01_Fab_‐Avaren was eluted with a linear gradient, utilizing MMC Buffer B (20 mm Tris‐HCl buffer + 1.5 m NaCl, pH 9) going from 500 to 1500 mm NaCl and pH 6 – 9 at a flow rate of 5 mL/min. Elution fractions were collected in 5 mL aliquots. VRC01_Fab_‐Avaren‐containing fractions (eluted between 25% MMC Buffer B and 70% MMC Buffer B) were again pooled and concentrated/buffer exchanged into CHT Buffer A (20 mm Tris‐HCl + 5 mm Sodium Phosphate, pH 8) using 30 kDa MWCO Amicon Ultra centrifugal filters. These were then loaded onto a CHT Type I column for the final purification step.

The 5 mL CHT column was equilibrated with 10 CV of CHT Buffer A at 5 mL/min. The prepared MMC elution (with the same conductivity to Buffer A) was loaded onto the column at 2.5 mL/min. Next a 10 CV wash with CHT Buffer A was performed to remove unbound proteins. A linear gradient using CHT Buffer B (20 mm Tris‐HCl + 5 mm sodium phosphate + 1500 mm NaCl, pH 8) ranging from 0% to 20% Buffer B over 20 CV was performed at 5 mL/min and the elution was collected in 5 mL fractions. The VRC01_Fab_‐Avaren‐containing fractions (eluted between 12% CHT Buffer B and 16% CHT Buffer B) were concentrated and buffer exchanged individually using 30 kDa MWCO Amicon Ultra centrifugal filters. Each fraction was visualized on a Coomassie Brilliant Blue (CBB)‐stained SDS‐PAGE gel and purity was determined using Carestream MI SE software after an image was captured on the Kodak Image Station 4000R Pro.

### Expression and production of control molecules, VRC01, Avaren‐Fc, VRC01_Fab_ and Av

Each of these molecules was expressed in *N. benthamiana* using the MagnICON^®^ tobamoviral vector system. VRC01_Fab_ and Avaren were each purified by a two‐step purification scheme consisting of Talon Immobilized Metal Affinity Chromatography (IMAC) from Clontech followed by CHT chromatography from BioRad. Production of VRC01 and Avaren‐Fc were done as described previously (Hamorsky *et al*., 2019; submitted).

### Coomassie Brilliant Blue (CBB)‐stained SDS‐PAGE and Western blot analysis

VRC01_Fab_‐Avaren along with the parental monomer molecules, VRC01_Fab_ and Avaren, were separated by SDS‐PAGE using the Mini‐PROTEAN TGX gels (12% Tris‐Glycine) on the Mini‐PROTEAN Tetra system from BioRad following manufacturer's instructions. The samples were prepared twice, under reducing and non‐reducing conditions (with and without 2‐mercaptoethanol). Once the proteins were separated (2 μg protein per lane), they were visualized using CBB stain. A separate set of gels were run for use in Western blot transfer (500 ng protein per lane for VRC01_Fab_‐Avaren and VRC01_Fab_; 250 ng protein loaded of Avaren). The Mini Trans‐Blot cell was used to transfer proteins from SDS‐PAGE to PVDF membrane at 100V for 45 min on ice. The blots were blocked using 5% (w/v) non‐fat dry milk in PBST (phosphate buffer saline with 0.05% Tween‐20) for 1 h. One blot was probed with goat anti‐human kappa chain HRP antibody (Southern Biotech #2060‐05) at a 1 : 3000 dilution in 1% milk/PBST for 1 h to detect the light chain of VRC01_Fab_. Another blot was probed with polyclonal anti‐actinohivin guinea pig serum at a 1 : 500 dilution in 1% milk/PBST for 1 h followed by incubation with goat anti‐guinea pig IgG HRP (Santa Cruz Biotechnology, Dallas, TX, USA) at a 1 : 1000 dilution to detect Avaren. Washes using PBST were performed between each step. The Western blots were developed using the GE Healthcare ECL Prime Western blotting detection reagent. Chemiluminescent images were captured using the Kodak Image Station 4000R Pro.

### Mannan‐coated ELISA

A mannan‐coated ELISA was utilized to detect VRC01_Fab_‐Avaren in elution fractions during purification. A clear flat bottom Maxisorp 96‐well plate (Thermo Fisher Scientific, Waltham, MA, USA) was coated with mannan (Sigma‐Aldrich, St. Louis, MO, USA) at 300 μg/mL (50 μL/well) in ELISA coating buffer (15 mm Na_2_CO_3_, 35 mm NaHCO_3_, 3 mm NaN_3_, pH 9.6) for 1 h at 37 °C. The plate was then blocked with 5% (w/v) milk/PBST (150 μL/well) for 1 h at 37 °C. Fifty microlitre of sample/well were placed on the plate in duplicates and incubated for 1 h and 37 °C. To detect VRC01_Fab_‐Avaren, a goat anti‐human kappa HRP antibody was placed in the wells at a 1 : 5000 dilution. The plate was washed three times with PBST between each of these steps. The plate was developed using a tetramethylbenzidine substrate (TMB) and ELISA stop solution (0.6 N H_2_SO_4_, 1 N HCl) and was read at 450 nm.

### Confirmation of bispecifity with ELISA

Wells of a 96‐well Maxisorp plate were coated with SIV gp120 (ImmuneTech Biotinylated SIVmac239 gp120 #IT‐001‐022‐Biotin) at 5 μg/mL concentration (50 μL/well) incubated overnight at 4 °C. Wells were blocked with 5% milk (w/v) in PBST (150 μL/well) for 1 h at 37 °C, followed by incubation with Avaren, VRC01_Fab_ and VRC01_Fab_‐Avaren, threefold serially diluted in duplicates starting at 10 μg/mL in 1% milk/PBST, for 1.5 h at 37 °C. In the wells containing monomer Avaren, anti‐actinohivin guinea pig serum polyclonal antibody was added at a 1 : 1000 dilution in 1% milk/PBST (50 μL/well). In the wells containing VRC01_Fab_ and VRC01_Fab_‐Avaren, an HRP‐conjugated anti‐human kappa antibody (Southern Biotech # 2060‐05) was added at a 1 : 500 dilution (50 μL/well). The plate was incubated at 37 °C for 1 h. After three washes with PBST, the portion of the plate incubated with anti‐human kappa antibody was developed using TMB substrate (50 μL/well) and ELISA stop solution (50 μL/well). The plate was then read at 450 nm. The Avaren portion of the plate was incubated with anti‐guinea pig HRP secondary antibody at a 1 : 1000 dilution in 1% milk/PBST for 1 h at 37 °C (50 μL/well). Then the plate was developed using the TMB substrate and the plate was read at 450 nm. Graphs were plotted in GraphPad Prism 5.0 software (GraphPad Software, San Diego, CA, USA).

### Mannosidase‐cleaved SF162 gp120 ELISA

Recombinant gp120 (SF162, HIV‐1/clade B, ImmuneTech #IT‐001‐0028p‐PBS) was treated with α(1‐2,3,6) mannosidase (PROzyme #GKX‐5010). A volume of 1 μL of gp120 (1 μg) was added to 10 μL of 5X reaction buffer (supplied by PROzyme), 20 μL of α(1‐2,3,6) mannosidase and 19 μL of Milli‐Q water, and the reaction mixture was incubated for 24 h at 37 °C. The mannosidase‐treated gp120 was plated in a 96‐well plate at 330 ng/mL in ELISA coating buffer (50 μL/well). The plate was then blocked with 5% milk (w/v) in PBST (150 μL/well). Threefold serial diluted proteins (Avaren, VRC01_Fab_ and VRC01_Fab_‐Avaren) starting at 10 μg/mL (50 μL/well) were applied to the plate and incubated for 1.5 h at 37 °C. The gp120‐bound Avaren was detected by the primary antibody anti‐actinohivin guinea pig serum at a 1 : 1000 dilution in 1% milk/PBST (1 h at 37 °C) and then secondary antibody goat anti‐guinea pig HRP (Santa Cruz) at a 1 : 1000 dilution in 1% milk/PBST (1 h at 37 °C). The gp120‐bound VRC01_Fab_ and VRC01_Fab_‐Avaren were detected by goat anti‐human kappa HRP antibody (Southern Biotech, Birmingham, AL, USA) at a 1 : 500 dilution in 1% milk/PBST (1 h at 37 °C). The plate was developed using TMB substrate and was read at 450 nm. PBST washes (3X per well) were performed between each step using an automated plate washer (ThermoScientific WellWash). This ELISA was repeated and provided the same results. A representative figure is shown. Graphs were plotted in GraphPad Prism 5.0 software.

### Bispecificity shown through surface plasmon resonance (SPR)

The binding affinity (*K*_D_) of the Avaren portion and the VRC01_Fab_ portion of the fusion, VRC01_Fab_‐Avaren, to gp120 was measured using the Biacore X100 2.0 instrument at ambient temperature. First, to determine the binding affinity of the VRC01_Fab_ portion of the fusion protein, mannosidase‐cleaved recombinant biotinylated gp120 (SF162, HIV‐1 clade B) was captured on a streptavidin (SA) sensor chip following manufacturer's instructions reaching a surface density of 162 response units (RU). A reference flow cell was utilized, which did not capture any gp120, to correct response contributions such as bulk shifts that occur equally in the sample and reference flow cells. Biotinylation of gp120 was done using the EZ‐LinkTM Sulfo‐NHS‐LC‐Biotinylation Kit (Vendor) following the manufacturer's instruction. To remove excess non‐reacted and hydrolyzed biotin remaining in the reaction, the sample was dialyzed against PBS, pH 7.4 using a 2 kDa MWCO 0.5 mL capacity Slide‐A‐Lyzer Dialysis Cassette (Thermo Fisher Scientific). Threefold serial dilutions of analyte, VRC01_Fab_‐Avaren, were made in running buffer (1X HBS‐EP supplied by GE Healthcare) and injected over the sensor chip at 5 μL/min, for a contact time of 120 s and dissociation time of 1000 s. To serve as controls, monomer Avaren and monomer VRC01_Fab_ served as analytes and were diluted and injected in the same fashion as the fusion protein. Between sample injections the sensor chip was regenerated with 10 mm Glycine, pH 3 for 30 s to remove any bound analyte and create a blank surface for the subsequent cycle. A blank cycle (running buffer) was performed and all sample injections were blank subtracted to correct the sensorgrams for drifts and other disturbances that affect the reference subtracted curve. A replicate of a non‐zero concentration of analyte and blank were injected in each experiment for double referencing thus verifying the reliability of the immobilized chip throughout the experiment. The data were analysed using a 1 : 1 binding kinetics fit with a parameter setting for *R*_max_ of ‘local’ and the offset of the RI set to zero in the Biacore X100 2.0 evaluation software.

Second, to determine the binding affinity of the Avaren portion of the fusion protein, VRC01_Fab_‐Avaren, recombinant biotinylated gp120 (SIV/mac239; Immune Technology, New York, NY, USA) was immobilized onto a SA sensor chip following manufacturer's instructions to a surface density of 1000 RU. Threefold serial dilutions of analytes, VRC01_Fab_‐Avaren, Avaren and VRC01_Fab_ were made in running buffer, injected at 5 μL/min for a contact time of 60 s and a dissociation time of 600 s. Between sample injections the sensor chip was regenerated with 10 mm glycine, pH 3 for 30 s to remove any bound analyte. A blank cycle (running buffer) was performed and all sample injections were blank subtracted to correct the sensorgrams for drifts and other disturbances that affect the reference subtracted curve. A replicate of a non‐zero concentration of analyte and blank were injected in each experiment for double referencing thus verifying the reliability of the immobilized chip throughout the experiment. The data were analysed using Steady State binding analysis in the Biacore X100 2.0 evaluation software.

### Pseudovirus production and HOS‐based env‐pseudotyped HIV‐1 neutralization assay

The antiviral activity of VRC01_Fab_‐Avaren, the parental monomers: VRC01_Fab_ and Avaren, and the parental dimers: VRC01 and Avaren‐Fc, was assessed based on a reduction in luciferase reporter gene expression after infection of HOS cells with Env‐pseudotyped viruses. The pseduoviruses were produced in 293T/17 cells as described (Matoba *et al*., 2010) using an Env‐expressing plasmid and an Env‐deficient HIV‐1 backbone vector (pNL4‐3.Luc.R‐.E‐). The Envs used in this study included CCR5‐tropic strains from clades A, B, and C: RW020.2, Q769.h5, SF162, QH0692.42 and ZM53M.PB12 obtained from the NIH AIDS Reagent Program. The viral infectivity level of the produced viruses was determined in HOS‐CD4‐CCR5+ cells as previously described (Hamorsky *et al*., 2019; submitted) to determine the optimal viral dilution yielding >150 000 RLUs. Samples and the virus were mixed and incubated for 1 h at 37 °C, to which 10 000 cells/well of HOS cells were added and incubated for 72 h. Luciferase activity was measured using the Luciferase Assay System (Promega Corporation, Madison, WI, USA). The antiviral activity of each sample was expressed as an IC_50_ value, which is the sample concentration giving 50% of relative luminescence units (RLUs) compared with those of virus control after subtraction of background RLUs.

## Conflict of interest

NM is an inventor on a patent related to the findings in this work (U.S. patent number: US8802822B2).

## Supporting information


**Figure S1** Breakdown of association and dissociation kinetics for Avaren, VRC01_Fab_ and VRC01_Fab_‐Avaren.
**Table S1** Average Combination Index Values for Avaren‐Fc and VRC01 at EC50, EC75 and EC90.
