# Supplementary material for: A novel anti‐HIV‐1 bispecific bNAb‐lectin fusion protein engineered in a plant‐based transient expression system
Source: Plant Biotechnol J. 2019 Mar 12;17(8):1646–56. doi: 10.1111/pbi.13090 (PMC6662308; doi:10.1111/pbi.13090)
Supplement: Supplementary file 1 — Figure S1 Breakdown of association and dissociation kinetics for Avaren, VRC01Fab and VRC01Fab‐Avaren. Table S1 Average Combination Index Values for Avaren‐Fc and VRC01 at EC50, EC75 and EC90. [file PBI-17-1646-s001.docx]

**Supplementary Information**

**TITLE:** A Novel Anti-HIV-1 Bispecific bNAb-Lectin Fusion Protein Engineered in a Plant-based Transient Expression System

**AUTHORS:** Lauren E. Seber Kasinger^1,5^, Matthew W. Dent^2,5^, Garima Mahajan^1^, Krystal Teasley Hamorsky^1,3,4^, Nobuyuki Matoba^1,2,3,*^

^1^James Graham Brown Cancer Center, University of Louisville School of Medicine, Louisville, KY, USA

^2^Department of Pharmacology and Toxicology, University of Louisville School of Medicine, Louisville, KY, USA

^3^Center for Predictive Medicine, University of Louisville School of Medicine, Louisville, KY, USA

^4^Department of Medicine, University of Louisville School of Medicine, Louisville, KY, USA

^5^These authors contributed equally to this work.

***Correspondence:** Nobuyuki Matoba, University of Louisville School of Medicine, 505 S. Hancock Street, Room 615, Louisville, KY 40202, USA, Tel: (502) 852 8412; Fax: (502) 852 5468; E-Mail: [n.matoba@louisville.edu](mailto:n.matoba@louisville.edu)


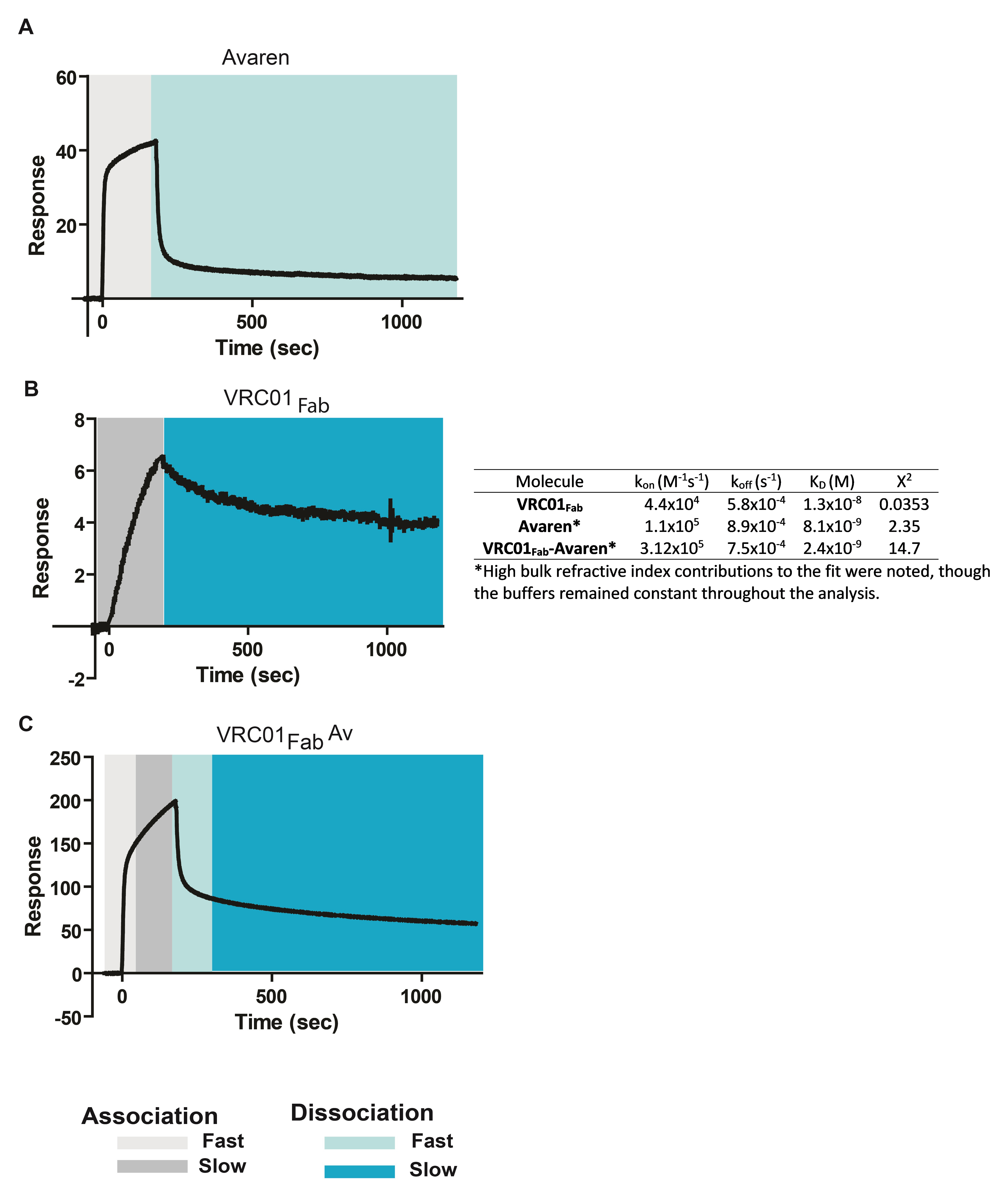


**Supplementary Figure 1 – Breakdown of association and dissociation kinetics for Avaren, VRC01_Fab_, and VRC01_Fab_-Avaren.**

Binding kinetics may explain the increase in activity of VRC01_Fab_-Avaren. As shown, Avaren has a slightly more rapid on-rate than VRC01_Fab_, with VRC01_Fab_-Avaren displaying an intermediate rate. Similarly, Avaren has a very rapid dissociation, while VRC01_Fab_ dissociates slowly and VRC01_Fab_-Avaren falls in the middle. We hypothesize that the Avaren moiety of the fusion binds to gp120 rapidly, giving the VRC01_Fab_ moiety more time to bind. With VRC01_Fab_ bound for a longer period of time, Avaren can dissociate and reassociate while being held in proximity to gp120, allowing for greater neutralizing ability.

**Supplementary Table 1 – Average Combination Index Values for Avaren-Fc and VRC01 at EC_50,_ EC_75_, and EC_90_.**

A CI of <0.9 indicates synergy, 0.9-1.1 indicates addition, and >1.1 indicates antagonism.

|  | Combination Indices (Mean ± SEM) | | |
| --- | --- | --- | --- |
| Virus | EC_50_ | EC_75_ | EC_90_ |
| **Q679.h5** | 0.25 ± 0.07 | 0.21 ± 0.06 | 0.23 ± 0.11 |
| **SF162** | 0.62 ± 0.13 | 0.43 ± 0.07 | 0.37 ± 0.13 |
| **ZM53M.PB12** | 0.30 ± 0.08 | 0.28 ± 0.03 | 0.38 ± 0.04 |
